# Antioxidant Activity and Multi-Elemental Analysis of Dark Chocolate

**DOI:** 10.3390/foods11101445

**Published:** 2022-05-17

**Authors:** Simona Jaćimović, Jelena Popović-Djordjević, Beka Sarić, Aleksandar Krstić, Violeta Mickovski-Stefanović, Nebojša Đ. Pantelić

**Affiliations:** 1 Institute of Field and Vegetable Crops, Maksima Gorkog 30, 21000 Novi Sad, Serbia; simona.jacimovic@ifvcns.ns.ac.rs; 2 Department of Chemistry and Biochemistry, Faculty of Agriculture, University of Belgrade, Nemanjina 6, 11080 Belgrade, Serbia; jelenadj@agrif.bg.ac.rs; 3 Department of Analytical Chemistry, Faculty of Chemistry, University of Belgrade, Studentski Trg 12-16, 11000 Belgrade, Serbia; beka@chem.bg.ac.rs; 4 Department of Physical Chemistry, Vinča Institute of Nuclear Sciences, National Institute of the Republic of Serbia, Mike Petrovića Alasa 12-14, 11000 Belgrade, Serbia; aleksandar.krstic@vin.bg.ac.rs; 5 Institute of Tamiš Pančevo, Novoseljanski Put 33, 26000 Pančevo, Serbia; mickovski.stefanovic@institut-tamis.rs

**Keywords:** cocoa, dark chocolate, polyphenols, flavonoids, major and trace elements, ANOVA, DPPH, FRAP, ICP-OES

## Abstract

Cocoa beans are part of the cocoa plant fruit (*Theobroma cacao* L.) used to prepare various products such as chocolate, cocoa butter, jelly, liqueurs, cosmetics, etc. Dark chocolate is consumed worldwide by different populations and is known for its good taste, making it one of the most favoured food products. This work aimed to determine the content of total polyphenols (TPC), total flavonoids (TFC), and the antioxidant potential measured through the ability to scavenge DPPH free radicals (DPPH), ferric reducing power (FRAP), and total antioxidant capacity (TAC), as well as major and trace elements contained in twelve commercially available dark chocolate samples, with cocoa content ranging from 40% to 99%. The total polyphenols content ranged between 10.55 and 39.82 mg/g GAE, while the total flavonoid content was from 10.04 to 37.85 mg/g CE. All applied antioxidant assays indicate that the sample with the highest cocoa percentage shows the greatest antioxidant activity (DPPH: 48.34% of inhibition; FRAP: 89.00 mg/g GAE; TAC: 83.86 mg/g AAE). Statistical methods were applied to establish the differences between the samples concerning TPC, TFC, DPPH, FRAP and TAC, as well as to differentiate the samples according to the mineral content. The results indicated that the differences in TPC and TFC between different samples depended on the cocoa content and the addition of dried fruit pieces. A good correlation between antioxidant potency composite index (ACI) and declared cocoa content was noticed (*R*^2^ = 0.8034), indicating that the declared percentage of cocoa is a reliable indicator for antioxidant activity of analysed dark chocolate samples. The nutritional evaluation proved that the studied chocolate samples were an excellent source of Mg, Fe, Mn and Cu.

## 1. Introduction

*Theobroma cacao* L. is a tropical crop of high importance, mainly due to the commercial value of its beans. Cocoa beans are part of the cocoa plant fruit. The botanical name of this plant in Latin means “Food of the Gods”, whereas the common name cocoa emerged from an Aztec language Nahuatl word xocolatl, which is derived from xococ (bitter) and atl (water) [1]. The cocoa tree is native to North Brazil, Colombia, Costa Rica, Ecuador, French Guiana, Guyana, Peru, Suriname, and Venezuela, but it was introduced to some tropical parts of Africa and Asia as well [1,2]. Nowadays, it is grown on a large scale on several continents (South America, Africa, and Asia), whereas in many countries worldwide, it is used for cocoa production, marketing, and consumption [2]. Moreover, the beans are used to prepare various products such as cocoa butter, chocolate, jelly, liqueurs, cosmetics, etc. Cocoa beans for chocolate production are obtained from three major varieties: Forastero, Criollo, and Trinitario [3], which grow in different tropical regions and produce cocoa beans with varying taste characteristics. Varieties grown in Central and South America (Trinitario and Criollo) produce the “fine” cocoas, which are distinguished by preferred aroma and colour, and are usually used for the production of dark chocolate [4,5].

Chocolate can be classified into dark, milk, and white, depending on manufacturing. In commercial dark chocolate, the solid cocoa content ranges from 47% (sweet dark) to 70%, 75%, or even above 90% for highly dark chocolate [6]. Nowadays, the trends in the chocolate industry are changing, influenced by increasing consumer concern with the nutritional status.

What is so unique about chocolate? In recent years, there has been growing interest in the study of natural components, primarily because of the consumers’ increased awareness that natural food ingredients are better and safer than synthetic ones. The primary nutrients of chocolate are fat, carbohydrates, and proteins [4]. In addition, chocolate contains various biologically active compounds such as polyphenols, lipo- and hydrosoluble vitamins, phospholipids, dietary fibers, and essential elements [5,6]. In contrast to vitamins and minerals, polyphenols are not essential compounds in human nutrition. However, they are consumed daily due to their omnipresence in foods. Due to the high level of polyphenols, as its main bioactive components, it has been documented that dark chocolate has possible positive effects on human health, e.g., regulating cholesterol levels, reducing stress and depression, even protecting against many types of cancer, etc. [7,8]. The most important polyphenol feature is attributed to the antioxidant activity [9,10,11,12,13,14]. This activity can be manifested by direct mechanisms such as neutralizing reactive oxygen species (ROS) or through indirect mechanisms that include activation of antioxidant enzymes, inhibition of pro-oxidative enzymes, and modulation of receptor-ligand interactions [15,16]. In addition, it is believed that dark chocolate has antiatherogenic, anti-inflammatory, and vasodilatory activities [17,18]. Furthermore, many studies have shown that it has a protective effect on erythrocyte function [19], against cardiovascular disease [20], and it can lower blood pressure in hypertensive subjects [21].

Major and trace elements are essential for the biological processes and the proper functioning of any living organism. Their supply depends on the bioavailability that is directly related to the composition of food [22]. Many studies have demonstrated that cocoa is a comprehensive source of many essential elements. Therefore, chocolate has the potential to provide a significant concentration of minerals in the human diet [23,24,25,26,27,28,29,30,31,32]. However, dark chocolate’s multi-elemental composition and bioactive compound content can vary depending on the conditions of cultivation and variety of cocoa beans, the conditions of their processing during fermentation and dying, as well as the chocolate-making process. Additionally, in chocolate production, pieces of dried fruit and vegetables are often added, which can influence its overall chemical composition [33,34].

This study aimed to investigate the quality of commercially available dark chocolate on the Serbian market, with the cocoa content ranging from 40 to 99 in percentage (%), in terms of the total polyphenol and flavonoid content, the antioxidant potential, as well as major-, and trace element content. Furthermore, given the importance of dark chocolate’s impact on consumers’ health, the correlation between the declared cocoa content and the antioxidant activity of the tested chocolate samples was evaluated. In addition, the samples were evaluated and compared to the nutritional reference values (NRV).

## 2. Materials and Methods

### 2.1. Materials

Commercially available dark chocolate and various international and national brands, with different content of cocoa, were analysed. Chocolate was purchased at local markets in Serbia, and the samples were labelled as follows: **S1** (40% cocoa with the addition of dried chili pepper, 32 g of fats, 60 g of carbohydrates, 5 g of proteins per 100 g), **S2** (49% cocoa, 31.8 g of fats, 51.1 g of carbohydrates, 4.9 g of proteins per 100 g), **S3** (56% cocoa, 36.2 g of fats, 45.1 g of carbohydrates, 5.8 g of proteins per 100 g), **S4** (73% cocoa, 47 g of fats, 23 g of carbohydrates, 8.8 g of proteins per 100 g), **S5** (75% cocoa, 40 g of fats, 33 g of carbohydrates, 10 g of proteins per 100 g), **S6** (75% cocoa with addition of dried orange, 34 g of fats, 39 g of carbohydrates, 8.3 g of proteins per 100 g), **S7** (75% cocoa with addition of dried raspberry, 38 g of fats, 35 g of carbohydrates, 9.5 g of proteins per 100 g), **S8** (77% cocoa, 45 g of fats, 24 g of carbohydrates, 8 g of proteins per 100 g), **S9** (77% cocoa with addition of orange, 46 g of fats, 25 g of carbohydrates, 9 g of proteins per 100 g), **S10** (85% cocoa, gluten-free, no sugar added, 49 g of fats, 19 g of carbohydrates, 11 g of proteins per 100 g), **S11** (88% cocoa, 58 g of fats, 15 g of carbohydrates, 10 g of proteins per 100 g), **S12** (99% cocoa, 49 g of fats, 10 g of carbohydrates, 14 g of proteins per 100 g). Five chocolate bars were taken (a total of 60 bars) for each of the twelve samples (**S1**−**S12**). Information about the producers and the nutrition facts of the analysed chocolate are given in Table S1 (Supplementary Materials). The solid chocolate was stored and kept in the refrigerator at 4 °C. Before the analysis, all the samples were chopped into small pieces. Each chocolate sample was prepared from randomly taken small pieces of five different bars.

### 2.2. Samples Preparation

In order to remove lipids, each of the analysed chocolate samples (5 g) was triturated with *n*-hexane (3 × 30 mL) at room temperature for 30 min. To evaporate organic solvent residues, degreased samples were air-dried for 24 h [35]. The defatted chocolate samples (1 g) were extracted three times with 10 mL of acetone-water-hydrochloric acid mixture (80:19.5:0.5%, *v/v/v*). Afterward, the samples were centrifuged at 4000× (TD4A-WS Benchtop Low-speed centrifuge, Shanghai, China) for 10 min, and the collected supernatants were evaporated (Heidolph, Rotary Evaporator, HB digit, Scwabach, Germany), transferred into a flask and filled up with extraction mixture acetone-water-hydrochloric acid, 80:19.5:0.5% (*v/v/v*) to obtain 10 mL of extract.

### 2.3. Determination of Total Polyphenol and Flavonoid Content

In order to determine the polyphenol and flavonoid content, the sample extracts were diluted 20 times to obtain the final concentration of 5 µg/mL samples. The content of total polyphenols (TPC) was determined according to the standard spectrophotometric Folin-Ciocalteu method [36]. Briefly, 0.1 mL of adequately diluted sample extract was mixed with 2 mL of Folin-Ciocalteu reagent, and after 3 min, 2 mL of 7.5% (*w*/*v*) Na_2_CO_3_ was added. After incubation for 2 h in the dark, the absorbance of the sample was measured at 765 nm (Shimadzu UV-1800 Spectrophotometer, Kyoto, Japan) relative to the blank sample. The concentrations were obtained using the calibration curve y = 0.0049x − 0.0188 (*R*^2^ = 0.9989). The results are expressed as mg gallic acid equivalent per gram of dry weight (dw) of the samples (mg/g GAE).

The total flavonoid content (TFC) of dark chocolate extracts was determined using the colorimetric assay with aluminium chloride described by Meng et al. [37] with slight modification. Briefly, 0.5 mL of adequately diluted sample extract was mixed with 0.15 mL of the 5% (*w*/*v*) NaNO_2_ solution. The content was shaken up on a Vortex (XH-D Vortex Mixer, Touch—Speed Adjustable, Jihan, Chine) and after 6 min 0.15 mL of 10% (*w*/*v*) AlCl_3_ was added. After 5 min, 1 mL of 1 M NaOH was added, then the solution was filled with water up to 5 mL, shaken up and the absorbance was measured on a UV-Vis spectrophotometer (Shimadzu UV-1800 Spectrophotometer, Kyoto, Japan) at 510 nm relative to the blank solution. The concentrations were obtained using the calibration curve y = 0.003x + 0.0084 (*R*^2^ = 0.9991). The results are expressed as mg catechin equivalents per gram of dry weight (dw) of the samples (mg/g CE).

### 2.4. Determination of Antioxidant Activity

***DPPH radical scavenging assay***: The antiradical activity of chocolate samples was determined using the free radical, 2,2-Diphenyl-1-picrylhydrazyl (DPPH), the method described by Brand-Williams et al. [38]. The working solutions were prepared by diluting the stock solution 40 times to obtain the final concentration of 2.5 µg/mL. Then, 10 µL of samples was added to 140 µL of DPPH radical solution (250 mM) and the tubes were kept for 20 min protected from light. Afterward, the absorbance was measured at 536 nm on a UV-Vis spectrophotometer (Shimadzu UV-1800 Spectrophotometer, Kyoto, Japan). The DPPH scavenging activity was expressed as a percentage of inhibition by using the following formula:

% of inhibition = [(*A*_control_ − *A*_sample_)/*A*_control_] × 100

***FRAP assay***: The ferric reducing antioxidant power (FRAP) assay was done using the method described by Bursal et al. [39] with slight modification. Shortly, the diluted extract solution (1 mL, 2.5 µg/mL) was mixed with phosphate buffer (1 mL, 0.2 M, pH 6.6) and potassium ferricyanide (1 mL, 1% (*w*/*v*)). The prepared solution was kept at 50 °C for 25 min in a water bath (Daihan, Digital Precise Water Bath, WB-22, South Korea), then the trichloroacetic acid (1 mL, 10% (*w*/*v*)) was added to the reaction mixture. A 1 mL aliquot of the mixture mentioned above was added to 1 mL of distilled water and 0.2 mL of 0.1% (*w*/*v*) ferric chloride, and the absorbance was measured at 700 nm (Shimadzu UV-1800 Spectrophotometer, Kyoto, Japan), after 10 min. The concentrations were obtained using the calibration curve y = 0.0065x − 0.033 (*R*^2^ = 0.9979). The results are expressed as mg gallic acid equivalents (GAE) per gram of dry weight (dw) of the sample.

***Total antioxidant capacity assay***: The total antioxidant capacity was evaluated spectrophotometrically using the phosphomolybdenum method [40]. An aliquot of the diluted sample solution (0.1 mL, 2.5 µg/mL) was shaken with 1 mL of reagent solution (0.6 M sulfuric acid, 28 mM sodium phosphate, and 4 mM ammonium molybdate). The mixture was incubated in a water bath (Daihan, Digital Precise Water Bath, WB-22, Seoul, South Korea) at 95 °C for 90 min. After cooling the samples, the sample’s absorption was measured at 765 nm (Shimadzu UV-1800 Spectrophotometer, Kyoto, Japan). The concentrations were obtained using the calibration curve y = 1.3451x − 0.028 (*R*^2^ = 0.9965). The antioxidant capacity was expressed as mg ascorbic acid equivalents (AAE) per gram of dry weight (dw) of the sample.

### 2.5. Antioxidant Potency Composite Index (ACI)

The antioxidant potency composite index was determined using the results obtained by antioxidant assays, as follows: ACI = sample score/best score × 100. The average of all tests for each chocolate sample was taken to calculate the final ACI value [41].

### 2.6. ICP Analysis

A total of twenty-six elements were analysed in DC samples: aluminium (Al), arsenic (As), barium (Ba), boron (B), calcium (Ca), cadmium (Cd), cobalt (Co), chromium (Cr), copper (Cu), iron (Fe), potassium (K), magnesium (Mg), manganese (Mn), molybdenum (Mo), sodium (Na), lead (Pb), nickel (Ni), sulphur (S), antimony (Sb), selenium (Se), silicon (Si), strontium (Sr), titanium (Ti), thallium (Tl), vanadium (V), and zinc (Zn), using the optical emission spectrometry with inductively coupled plasma (ICP-OES) technique.

The chocolate samples were crumbled and prepared by acid wet digestion. The mass of 1 g of each sample was weighed in glass vessels and heated with 10 mL concentrated HNO_3_ (65%, *v*/*v*, Zorka, Šabac, Serbia) for 30 min at 80 °C. Then, 5 mL of H_2_O_2_ (30%, *v*/*v*, Zorka, Šabac, Serbia) and 5 mL concentrated HNO_3_ (65%, *v*/*v*, Zorka Šabac, Serbia) were added and heated for another 90 min at 80 °C to complete digestion. Finally, the solutions were cooled, filtered through quantitative filter paper (pore diameter was 0.22 µm), transferred to a volumetric flask (50 mL), and diluted to the mark with ultrapure water.

The element content was determined by Thermo Fisher Scientific (Waltham, MA, USA) model 7400 duo ICP-OES spectrometer [42,43,44]. Qtegra Intelligent Scientific Data Solution (ISDS) from Thermo Fisher Scientific was used for data processing. The analytical standard for the instrument calibration was prepared by one multi-element standard solution (Multi stock: Certipur ICP multi-element standard solution IV, HC67834755, 1000 ppm) and one mono-element Hg standard solution (Mercury solution, 1402980, 1000 ppm, Sigma-Aldrich Chemie GmbH, Munich, Germany). The working standard solution was prepared by subsequent serial dilution of stock standards. All experiments were performed at room temperature (25 °C). The blank solution was prepared in the same manner as the samples. Method parameters and method detection limits (MDL)/method quantification limits (MQL) for elements are presented in Tables S2 and S3, respectively (in Supplementary Materials).

### 2.7. Dietary Intake of Nutritional Elements

Dietary intake (nutritional contribution) of essential elements (K, Ca, Mg, Fe, Mn, Cu, and Zn) from 100 g of dark chocolate was calculated based on the concentrations of elements, compared with nutritional reference values (NRV) and expressed in percentage. NRV values are established by national legislation [45].

### 2.8. Statistical Analysis

Principal Component Analysis (PCA) was applied using PLS ToolBox, v.6.2.1 (Eigenvector Research, Inc. 196 Hyacinth Road Manson, WA, USA), for MATLAB (7.12.0 (R2011a)) (http://www.eigenvector.com/software/pls_toolbox.htm, accessed on 20 October 2021) Research, Inc., Wenatchee, WA, USA). PCA was carried out as an exploratory data analysis using a singular value decomposition algorithm (SVD) and a 0.95 confidence level for Q and T2 Hotelling limits for outliers. The PCA grouped the parameters based on their similarity and resulted in a lower number of principal components that, in turn, reduced the dimensionality of the retention data space, thus further simplifying the analysis [46].

Descriptive statistics, correlation coefficients, and differences between the mean values of different methods (total polyphenol content (TPC), total flavonoid content (TFC), DPPH, ferric reducing antioxidant power (FRAP), and total antioxidant capacity (TAC)) were obtained using Microsoft Office Excel 2010 (data analysis). To confirm the existence of statistically significant differences between the samples within one method, Tukey’s test was applied using a demo version of NCSS statistical software [47]. All data were reported as the mean of three measurements.

## 3. Results and Discussion

Descriptive statistics (mean, standard deviation, median, minimum, and maximum values) provided information on the content of total phenols (TPC) and total flavonoids (TFC) using different antioxidant assays in dark chocolate (40−99% of cocoa), as shown in Table 1.

### 3.1. Total Polyphenol and Total Flavonoid Content

Total polyphenol and total flavonoid content (TPC and TFC, respectively) of chocolate extracts (**S1**−**S12**) were evaluated spectrophotometrically, and the results are presented in Table 1. According to literature data, the total content of polyphenols and flavonoids varies greatly depending on the polyphenols’ extraction method and the type of used solvent [48,49,50]. The acidified aqueous acetone solvent proved to be the most efficient extraction agent for the extracting of polyphenolic compounds from chocolate [51]. Therefore, to achieve the highest efficiency in the content of mentioned bioactive components, the chocolate extracts in this study were obtained by extracting the chocolate samples with acetone-water-hydrochloric acid (80:19.5:0.5%, *v*/*v*/*v*) as an extraction agent.

The results showed that TPC ranged between 10.55 and 39.82 mg/g GAE. The obtained results demonstrated that the sample with the highest cocoa percentage exhibited the highest polyphenol content. In addition, the samples of the dark chocolate with dried orange or raspberry pieces contained a greater amount of polyphenols than the chocolate samples without the addition of fruit with the same cocoa content. Therefore, by observing the obtained results, it can be concluded that there are differences in the amount of polyphenols in different chocolate samples, depending on the amount of cocoa in dark chocolate and the addition of dried fruit pieces. The study conducted by Waterhouse et al. [52] shows the result of 20 mg/g GAE for cocoa powder, using 95% aqueous methanol as the extraction solvent. This result is lower than the results obtained in this study. Still, it is known that the total polyphenol content varies depending on the cocoa bean variety and the conditions during fermentation, drying, roasting, processing, and storage [53]. Furthermore, the obtained results for TPC are in accordance with the data obtained in the study on dark chocolate samples [54,55]. On the other hand, in some cases, obtained results cannot be directly compared due to the differences in the applied extraction solvent. Moreover, some literature results are presented as mg of standard compound per gram of cocoa product, while the other results are presented as mg of standard compound per gram of defatted product from the cocoa product, which contributes to some deviations between the results [49].

Analysis of variance (ANOVA) was performed within each method individually to detect the differences in the chocolate samples with different cocoa content and different fruit or vegetable additives. Within one method applied to different samples, the variance analysis showed a statistically significant difference and the post hoc Tukey’s HSD test confirmed it. The individual samples within one method differed significantly statistically but we proved which ones exactly led to the difference (Table 1) and the difference was between the samples with a different letter next to the value of mean ± SD (Table 1). Samples **S9** and **S10** and samples **S2**, **S3**, **S4,** and **S5** had values followed by the same letter, which means that they did not differ significantly (*p* < 0.05), according to Tukey’s HSD test. Sample **S9** showed no difference compared to sample **S10**. Samples **S2**, **S3**, **S4,** and **S5** had the percentage of cocoa ranging from 49% to 75% and there was no significant difference in TPC. Statistically significant differences were observed for all other samples.

Furthermore, the results showed that the flavonoid content in the samples ranged from 10.04 to 37.85 mg/g CE. The TPC was similar to the obtained results, which indicated that the sample with the highest percentage of cocoa showed the highest flavonoid content. Again, the samples of the dark chocolate with dried pieces of orange or raspberry showed a slightly greater amount of flavonoids compared to the samples of chocolate without the addition of fruit with the same cocoa mass content. Thus, by observing the obtained results, it can be concluded that there are differences in the TFC for different samples of studied chocolate, depending on the cocoa amount in the dark chocolate and the presence of dried fruit pieces. The contribution of powdered fruit and vegetables to chocolate’s phenolic content and antioxidant activity has recently been reported in the literature [33], which supports the results of the present study.

The TFC accounts for an average of 98% of the total polyphenol content, indicating that this group of bioactive compounds predominates in cocoa polyphenols. The content of TFC is notably higher than what other authors obtained, which can be explained by the applied extraction agent [56]. According to previous investigations, water is less efficient than acetone, methanol, or ethanol as a solvent for extracting bioactive compounds from chocolate [49,54].

ANOVA was performed within each method individually to detect the differences in chocolate samples with different cocoa content (with or without fruit or vegetables). According to the post hoc Tukey’s HSD test (which shows the difference based on the different letters in Table 1), neither the TFC nor TPC differed significantly between the samples **S9** and **S10**. Also, the **S7**, **S6**, **S4,** and **S2** samples did not differ from each other. There was also no statistically significant difference between the samples **S6**, **S4**, **S3,** and **S2**, or **S5**, **S4,** and **S3** (Table 1).

Correlations between the content of total polyphenols and total flavonoids were examined using Pearson’s correlation (Table 2). In addition to the excellent correlation between TPC and TFC (r = 0.99), positive high correlations were noticed for TFC, FRAP (r = 0.98), TFC and DPPH (r = 0.92) (Table 2).

### 3.2. Antioxidant Activity of Dark Chocolate Samples

Since cocoa’s physiological and health-related effects are important to consumers, the antioxidant capacity of dark chocolate with different cocoa content available on the Serbian market was evaluated by performing different assays. Many studies show that the radical system used to assess antioxidant activity can influence experimental results. Bearing this in mind, at least two methods are required to evaluate the ability of the analysed sample to trap the radicals. Consequently, in this study, DPPH (2,2-diphenyl-1-picrylhydrazyl), FRAP (ferric reducing antioxidant power), and TAC (total antioxidant capacity) assays were used to assess the antioxidant activity of the extracts obtained from the dark chocolate samples with different cocoa percentage. Although the experiments were performed at a very low concentration of the samples (2.5 µg/mL), the obtained results have shown a high antioxidant potential, Table 1.

**DPPH radical scavenging assay:** The results obtained using the DPPH method indicate that the percentage of cocoa in the analysed samples influenced the antioxidant activity of dark chocolate (Table 1). At the applied concentration of the samples (2.5 µg/mL), the lowest ability to scavenge DPPH free radicals was observed in **S1** (29.61 ± 0.70% of DPPH inhibition), while the greatest ability was detected in the sample with the highest percentage of cocoa (**S12**, 48.34 ± 0.99% of DPPH inhibition). It can be noticed that the samples with lower polyphenol and flavonoid content showed lower antioxidant activity. DPPH inhibition of the samples enriched with fruit (**S6, S7,** and **S9**) did not significantly differ from the samples with fruit and the same cocoa content (**S5** and **S8**), which corroborates the results of Żyżelewicz et al. [33].

Correlations between different antioxidant assay methods were examined using Pearson’s correlation (Table 2). DPPH showed a good correlation with TPC, as well as DPPH with FRAP (r = 0.93). Unlike DPPH, TAC showed the lowest correlation (r = 0.32), Table 2. After the ANOVA for the DPPH method was performed to detect differences in chocolate samples with different cocoa content (with and without fruit or vegetables) and to obtain statistically significant differences, a post hoc Tukey’s HSD test was performed showing similarity between the samples with the same letters (Table 1). The samples with the highest percentage of cocoa, **S11** and **S12**, didn’t differ statistically. The samples **S10**, **S9**, and **S8** also didn’t differ from each other. Additionally, the samples with a low cocoa percentage (**S2** and **S3**) did not differ from each other according to DPPH inhibition. Furthermore, dark chocolate sample **S6** (with dried pieces of orange) did not show a statistically significant ability to inhibit DPPH solution compared to the chocolate samples **S4** and **S5** with similar or the same cocoa content and without fruit.

**Ferric reducing antioxidant power (FRAP) assay:** The obtained results showed that the antioxidant activity ranged between 20.50 and 89.00 mg/g dw GAE (Table 1). The sample with the highest percentage of cocoa (**S12**) showed the highest antioxidant capacity, which is in line with the results obtained by the DPPH test. So far, the results in the literature showed the effect of the ingredients added to chocolate on its antioxidant capacity. The studies conducted by Todorović et al. [54] have demonstrated that even though there is no significant difference in the total polyphenol and flavonoid content between dark chocolate and dark chocolate with a small amount of added raspberries, a substantial increase in antioxidant activity may be due to other ingredients with a high antioxidant potential present in raspberries [54]. Furthermore, a similar conclusion was drawn by analysing the dark chocolate samples with the same cocoa mass content with and without rosemary. The sample with rosemary exhibited higher antioxidant activity, most likely due to the compounds present in rosemary, such as rosmarinic acid, carnosic acid and carnosol [57]. As far as FRAP assay is concerned, a statistically significant difference was observed in the samples with a high percentage of cocoa, with or without fruit, **S8**, **S9**, **S10, S11** and **S12**. Additionally, there is a statistically significant difference between the samples with the same percentage of cocoa mass and added dried pieces of fruit (**S6**, **S7**), but also compared to the sample without dried pieces of fruit (**S5**). Similarity, statistical significance was shown in samples **S3**, **S4**, and **S5** as well as **S2**, **S3**, and **S5**.

**Total antioxidant capacity (TAC) assay***:* The phosphomolybdate method is based on the spectrophotometric quantification of total antioxidant capacity (TAC). In accordance with the results obtained by the DPPH and FRAP assays, the sample with the highest percentage of cocoa (**S12**) showed the highest antioxidant capacity (83.86 ± 1.6 mg/g AAE), Table 1. Samples **S12**, **S11,** and **S2** didn’t differ among themselves (Table 1). However, sample **S2** showed significant deviations and an unexpectedly high value of antioxidant capacity, probably due to the influence of cocoa used to produce this sample of chocolate (origin, variety, etc.). In addition, samples **S3**, **S9**, and **S10** showed similarities, which is also the case with samples **S1** and **S8**. Reasons for the observed irregularities can be associated with orange and paprika as antioxidant-rich ingredients or with the geographical origin of the used cocoa beans and manufacturing process, which could affect the total antioxidant capacity of chocolate. It can be observed that increasing the percentage of cocoa mass in the samples increases the antioxidant capacity. In addition, dark chocolate sample **S7** with raspberry pieces had higher antioxidant activity, while orange pieces in sample **S6** did not increase the antioxidant activity compared to sample **S5** with the same cocoa mass and without fruit.

All applied methods were compared with a one-way ANOVA to test statistically significant differences between the mean values of these methods (TPC, TFC, DPPH, FRAP and TAC) using Microsoft Office Excel 2010 (Data Analysis), Table 1. The differences between mean values at the level of 5% (*p* < 0.05) are considered close, and ANOVA showed that there is no significant difference between the studied parameters (TPC, TFC, DPPH, FRAP and TAC) for the Area parameter at *p* < 0.05 (F_calculated_(1.6) < F_critical_(2.0)). The absence of statistically significant differences between them can be considered proof that the measurements belong to one large group of methods, which we have previously shown by correlation (Table 2).

**Figure 1:**
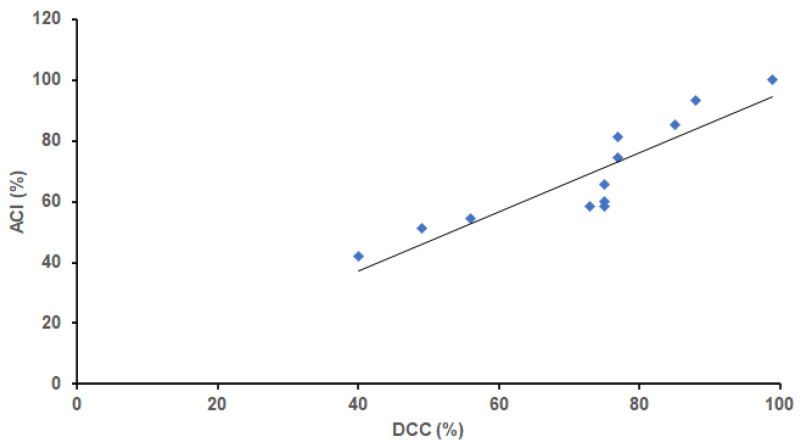
Correlation between antioxidant potency composite index (ACI) and declared cocoa content (DCC) in analysed chocolate samples.

Since the content of polyphenolic compounds depends mainly on the cocoa content in chocolate [54], the correlation between declared cocoa content and the antioxidant activity of analyzed samples was evaluated through the antioxidant potency composite index (ACI). As a result, a good correlation between calculated ACI values and declared cocoa content (*R*^2^ = 0.8034, Figure 1) was noticed, indicating that the information on cocoa percentage in dark chocolate samples is a reliable indicator of their antioxidant activity [54]. Besides, consuming chocolate with very high cocoa content (**S10**, **S11**, and **S12**) results in lower carbohydrate (<20 g per 100 g of sample) and higher protein intake (11, 10 and 14 g per 100 g of sample, respectively), so in terms of these nutrients, the mentioned chocolates are of better quality than those with a low cocoa content.

### 3.3. Multi-Elemental Analysis

The successful determination of elements in chocolate is still a challenge due to the characteristics of its complex matrix. Namely, chocolate has a high content of organic compounds (e.g., hydrogenated vegetable oil, vegetable fats, solids from malt extract), salts, emulsifiers, etc., which makes the appropriate decomposition or digestion of the sample(s) difficult [31]. The occurrence of 25 elements and their concentrations in the dark chocolate samples was assessed by the optical emission spectrometry with inductively coupled plasma (ICP-OES) technique. Seventeen elements (Al, Ba, Ca, Cd, Co, Cr, Cu, Fe, K, Mg, Mn, Ni, S, Si, Sr, Ti, and Zn) were quantified in all the samples, with the exception of Si in the samples **S4**, **S9** and **S10**. Concentrations of elements such as As, B, Mo, Pb, Sb, Se, Tl, and V were below the method quantification limits (MQL) in all the studied samples, whereas Na was quantified in only three samples (**S5**−**S7**).

Parameters of descriptive statistics for the elemental profile of chocolate with different cocoa content (40−99%) are presented in Table S4 (Supplementary Materials). According to the obtained data, mean concentrations of major elements (mg/kg) in the chocolate samples were in the following ranges: 337.69−908.12 (Ca), 695.3−2962.1 (K), 663.48−1564.86 (Mg) and 1053.6−3009.7 (S). Among trace elements, the most abundant was Fe with mean concentrations of 56.9−478.95 mg/kg. Other elements of this group (Al, Ba, Cd, Co, Cr, Cu, Mn, Ni, Si, Sr, Ti, and Zn) were measured in concentrations less than 40 mg/kg in all the studied samples (Table S4). The lowest and the highest concentrations were observed for Cd (0.03 mg/kg in **S9**) and Al (36.19 mg/kg in **S5**), respectively. Elements such as As, Ba, Be, Bi, Cr, Ni, V, and Tl are not desirable in chocolate, as they are characterized as toxic or potentially toxic elements [25]. Some literature outlines Al, Ba, Fe, Co, Cr, and Mo as possible contaminants in cocoa beans. On the other hand, they could arise from industrial processes during the preparation of chocolate [28].

Multi-elemental analysis of dark chocolate with different cocoa content is reported in the literature, and the data obtained from several studies that used the ICP analytical method are presented in Table S5 (Supplementary Materials). In addition, the data from the recent study where Microwave-induced plasma optical emission spectrometry (MIP-OES) as a new and promising method was used for multi-elemental analysis of chocolate, were included too, as the results are comparable to those obtained by the ICP analysis [32].

#### 3.3.1. Major Elements

The mean concentration of Ca in the dark chocolate samples ranged from 337.68 mg/kg in **S2** to an almost three-fold higher concentration in **S6** (908.12 mg/kg). The obtained results were in line with the results reported in the literature [28,29,30,32]. On the other hand, higher values for the content of Ca in dark chocolate were reported by other authors [25,27,31].

Potassium (K) was measured in concentrations of 695.3−2962.1 mg/kg in samples **S2** and **S12**, respectively, which is substantially lower compared to the data [27,28,29,30,32]. Only Karaś et al. [31] reported a somewhat lower content of K (381.9−1082.7 mg/kg). The content of K in the studied chocolate was gradually increasing with the increasing cocoa content, but it was interesting that the samples without the addition of fruit had a higher content of K when compared to the samples with pieces of fruit, despite having the same percentage of cocoa: **S5**, **S6** and **S7**, and **S8** and **S9**.

The content of Mg in the studied chocolate ranged from 663.48−1564.86 mg/kg; the lowest/highest content was in the plain dark chocolate with the lowest (**S2**) and the highest (**S12**) cocoa content. The results were in line with those obtained by Mrmošanin et al. [29], whereas other literature data suggest higher concentrations of this element in dark chocolate [25,27,28,30,32].

The content of S (1053.6−3009.7 mg/kg) in the studied samples was 2–5-fold higher compared to the results reported by other authors [25,31]. Chocolate with 56% of cocoa (**S3**) had the lowest, while the richest cocoa chocolate (**S12**) had the highest S content.

#### 3.3.2. Trace Elements

The content of Al ranged from 11.38−36.19 mg/kg, in samples **S3** and **S5**, respectively. The obtained results were in line with some literature data [25,29,31], while Cinquanta et al. [28] reported a lower content of this element. Although Al could be found in silver packaging foil for chocolate, it is unlikely to be a source of Al contamination [25]. For adults, the tolerable daily intake of Al is 1 mg/kg of body weight [58]. Barium content in the studied chocolate varied between 1.69 mg/kg (**S2**) and 4.36 mg/kg (**S12**). Barium is a non-essential element available to humans from sources such as water and food. In sweets and confectionary its concentration is 0.045−5.10 mg/kg [59]. The obtained results agreed with the literature data for Ba in dark chocolate [29,31], whereas the other authors reported a higher content [25,28].

Cobalt is a part of the prosthetic group in hem-compound cobalamin, vitamin B_12_, one of the most chemically complex of all vitamins [22]. The content of Co in cocoa and cocoa-based products are reported to be 0.92 and 0.093 mg/kg, respectively [25]. In the studied chocolate, Co content was below 0.5 mg/kg, except the samples **S11** and **S12**, in which it was measured in slightly higher concentrations. The highest/lowest concentrations were in the samples **S11** (0.53 mg/kg) and **S2** (0.19 mg/kg), respectively. These results were in line with the literature [25,28,29].

Chromium in the form of chromate (Cr(VI)) is accumulated in plants and interferes with sulphate metabolism by sulphate-uptake systems. When Cr(VI) is reduced to Cr(III) ion, radicals are formed, making Cr a toxic and carcinogenic element. Chromium was classified as an essential element in the past because this element binds to peptides, and such a complex has a role in insulin metabolism in humans [22]. However, new findings oppose this classification [60]. Tolerable daily intake for Cr is 3.5 μg/per kg of body weight [58]. Concentrations of Cr were measured from 0.07 mg/kg in **S9** to 2.63 mg/kg in **S12**. The results corroborated the literature data [6,25,29,30,32]. Although a positive linear correlation between Cr concentration and cocoa content in chocolate was observed by some authors [6], such correlation was not found in this study.

The concentration of Cu in the dark chocolate varied between 6.70 mg/kg (**S3**) and 17.14 (**S12**), which was in line with the literature [25,26,27,28,29,30,32], whilst substantially higher concentrations of Cu were reported by Karaș et al. [31]. In addition, some authors reported a linear correlation between cocoa and Cu content [26], but no such correlation was observed in this study.

The range of Fe content was from 56.9 mg/kg (**S9**) to 478.95 mg/kg (**S12**). The obtained results were in line with the literature, although the sample **S12** had a substantially higher amount of Fe than was reported in the literature [27,28,31,32]. On the other hand, Grassia et al. [30] reported a 5–10-fold lower content of Fe compared to its content in all the chocolate samples studied here.

Manganese naturally occurs in many foods, including cocoa and chocolate, rich sources of this element. In cocoa it may reach 38.8–53.4 mg/kg [25]. In the dark chocolate studied here, the content of Mn was 6.9−21.52 mg/kg (samples **S9** and **S7**, respectively), which fully corroborated the data in the literature [25,27,28,29,31,32].

The content of Ni in the chocolate samples was in the range of 1.62 to 4.94 mg/kg. In comparison to other food items, such as vegetables, Ni content was quite higher. In general, the results were in line with the literature [25,28,29,31]. However, slightly higher values were reported by authors who analysed Ni in chocolate samples using MIP-OES [32]. A higher content of Ni may be due to the chocolate preparation process or from the equipment containing Ni [25].

Although silicon is not considered an essential element for humans, it is important for bone calcification. It helps the wound healing process and is important for the optimal synthesis of collagen, a protein found in various connective tissues [61]. Concentrations measured in the chocolate samples varied from MQL (0.28 mg/kg, **S4**, **S9**, **S10**) to 5.71 mg/kg (**S12**), which is substantially lower than what was reported in the literature [25,29]. Strontium is distributed in the human body in a similar way to Ca and is mostly found in the skeleton. It stimulates bone growth and increases bone density. Strontium-based medication (strontium ranelate) is helpful in the treatment of postmenopausal osteoporosis [59]. The content of Sr varied from 3.7−8.76 mg/kg measured in the samples with the lowest and the highest cocoa content (**S1** and **S12**, respectively). The obtained results were in accordance with the results of other authors [25,29,31].

The content of Ti was in the range from 0.08 mg/kg (**S6**) to 1.64 mg/kg (**S12**). The study conducted by Sager [25] reported significantly lower concentrations of Ti in dark chocolate. Food is a major source of Ti for humans. Absorption upon ingestion of titanium compounds is generally low. It makes Ti relatively non-toxic, in addition to being a non-essential element [59].

The obtained results for the content of Zn in the studied dark chocolates, which ranged from 12.15 mg/kg (**S2**) to 34.57 mg/kg (**S12**), were supported by the literature [25,28,29,30,31,32], whereas a two-fold higher content was reported by Villa et al. [27].

Two elements, cadmium and lead, are of special concern when cocoa and chocolate with a high cocoa content are studied. An elevated content of Cd may be present in marketed chocolate and cocoa powder, and in this regard, they are an important source of human exposure [62]. This is even more pronounced, considering that the bioavailability of Cd from cocoa products (powder, beans, liquor, etc.) may be up to 50% [63]. Cadmium is a highly toxic element, that primarily accumulates in the kidneys and is slowly eliminated from the body [64]. The presence of Cd may be from both natural and anthropogenic sources; either the plant takes it from the contaminated soil, or the contamination occurs during the manufacturing process [65]. The European Commission has revised (applied since 1 January 2019) the maximum permissible level (ML) for Cd in cocoa powder and chocolate, and for chocolate with ≥50% total dry cocoa solids, it is set at 0.8 mg/kg [62].

**Figure 2:**
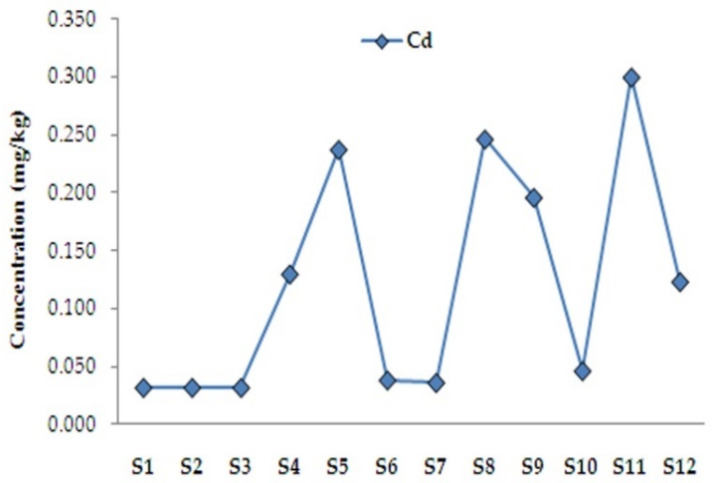
Distribution of Cd within the samples; cocoa content: **S1**—40% with pepper, **S2**—49%, **S3**—56%, **S4**—73%, **S5**—75%, **S6**—75% with orange, **S7**—75% with raspberry, **S8**—77%, **S9**—77% with orange, **S10**—85% (gluten-free, no added sugar), **S11**—88%, **S12**—99%; (*n* = 3).

The obtained results indicated that with the increase of cocoa percentage in the samples, Cd concentration also increased, with the exception of the samples with fruit components (**S6**, **S7**, **S9**), Figure 2. Indeed, evaluation of cadmium concentration in chocolate products continues to be an analytical challenge if the matrix complexity is taken into consideration. Suitable sample treatment with a sensitive analytical technique is a prerequisite for a successful analysis [27,31]. What is more, two samples, one with a high percentage of cocoa but with no added sugar (**S10**) and another with 99% of cocoa (**S12**), also showed irregularity in the correlation between Cd concentration and cocoa percentage (Figure 2). The published studies report dissimilarity in the relative content of Cd in chocolate (Table S5), which may be attributed to different factors, including the environmental sources of Cd and Pb and the geographic origin of the cocoa beans [25,29,65]. A survey by Abt et al. [65] indicated that the content of Cd was dependent on the percentage of cocoa in chocolate samples, and it increased with the increase of cocoa content in chocolate. However, in the present study, the concentrations of Pb and Cd were below the MQL and within the ML set by the European Union regulations, respectively. So far, no data on the content of Cd in dark chocolate with fruit are available in the literature, so these results give a new insight into the analysis of such products. Nonetheless, it could be assumed that the origin of cocoa used for the production of the studied chocolate and manufacturing practice are the most likely reasons for such results.

An ANOVA was performed for the content of Cd. It showed that there was a statistically significant difference between the samples with different cocoa percentages. In addition, a post-hoc Fisher test—LSD was also performed, which confirmed that the differences in the mean values of the samples **S1**−**S2**, **S1**−**S3**, **S2**−**S3,** and **S6**−**S7** led to statistically significant ones (the values of the differences between the samples themselves are higher than the values of LSD (0.0033) (Supplementary Materials Table S6).

In general, concentrations of elements varied between the chocolate samples depending on the cocoa content. The highest content for most of the analysed elements (Ba, Cr, Cu, Fe, K, Mg, Ni, S, Si, Sr, Ti, and Zn) was found in sample **S12**. On the other hand, sample **S2** had the lowest content of Ba, Ca, Co, K, Mg, Sr, and Zn. These findings are consistent with the literature data. Namely, Karaś et al. [31] reported the highest content of Ba, Cu, Mg, Mn, Ni, P, S, Sr, and Zn in dark chocolate with 90% of cocoa.

### 3.4. Dietary Intake of Nutritional Elements

Major and trace elements (Ca, Mg, K, P, Cu, Mn, Fe, Zn, Mo, Se, I) are regarded as important elements for human nutrition. Although they are not consumed in large quantities, the normal functioning of a living organism depends on these elements and, therefore, must be regularly present in the diet [66]. As a cocoa-based product, dark chocolate is naturally rich in polyphenols, flavonoids, and elements such as Mg, Fe, Mn, and Cu. The contribution of each element to NRV for adults (over 30 years of age) set by national legislation was determined based on the content of elements in 100 g portion of the studied dark chocolate samples [45]. Results are presented in Figures S1 and S2 (Supplementary Materials).

The obtained results suggest that chocolate (40−99% of cocoa) may contribute to NRV of K and Ca with 4.2−11.4% and 3.5−14.8%, respectively. A lower contribution of both elements is observed in chocolate with a cocoa content of 40−56% (**S1**−**S3**). Chocolate samples with added fruit offered less K compared to plain chocolate samples with the same cocoa content. The highest contribution of the studied chocolate to NRV was observed for Mg. Most of the chocolate samples contributed more than 20%. The sample with the highest cocoa content (**S12**) stood out with 41.7% (Figure S1).

For trace elements, the contribution of chocolate to NRV was more pronounced, and for Fe, Mn, and Cu in all the samples, it was over 30%. The percentage of Fe, Mn, and Cu dietary intake from 100 g of dark chocolate compared to NRV was in the ranges: 40.7−342.1, 34.5−107.6, and 67−171.4, respectively. Indeed, 100 g of chocolate may contribute to 50% of Cu daily recommended intake, and those with higher cocoa percentage even more [26]. Substantially lower dietary intake was observed for Zn, 12.2−34.6% (Figure S2).

Literature data indicate that the bioaccessible fractions in chocolate drink powder are: Mg > 50%, Fe < 5%, Zn 15−30%, Mn 11%, and Cu 29%. Dietary components such as proteins, peptides, amino acids, polysaccharides, sugars, lignin, phytin, and organic acids may influence the absorption of nutritional elements [24]. Although dietary intake of nutritionally valuable elements via consumption of the studied chocolate may be important, (Figures S1 and S2), it is also crucial to have the data on their bioaccessibility to estimate the real benefits.

### 3.5. Principal Component Analysis (PCA) and Hierarchical Cluster Analysis (HCA)

A principal component analysis (PCA) was used to establish the differences between different chocolate samples. Analysis of the main components based on the content of 17 elements (Al, Ba, Ca, Cd, Co, Cr, Cu, Fe, K, Mg, Mn, Ni, S, Si, Sr, Ti, and Zn) in the chocolate samples resulted in a three-component model that explained 87.46% of the total variability among the data. The first principal component accounted for 70.01%, the second 9.53%, and the third component 7.92% of the total variance. Statistical parameters are shown in Table 3. All data were autoscaled prior to any multivariate analysis.

**Figure 3:**
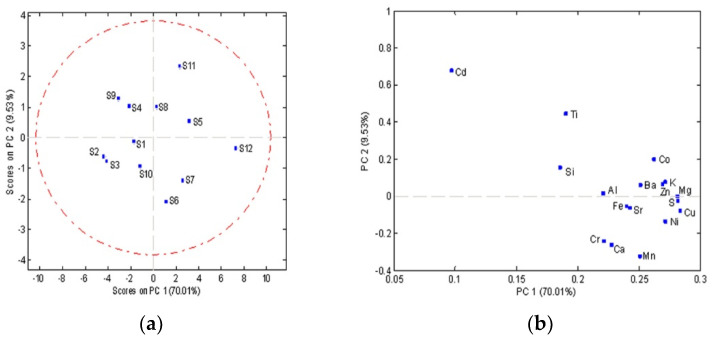
Analysis of the main components based on the content of 17 elements in chocolate samples; (**a**) Score plots of the first two principal components; (**b**) loading plots.

Mutual projections of factor scores and their loading for the first two PCs for 17 minerals are shown in Figure 3. The separation of the samples into two groups can be observed along the PC1 axis. As it can be seen from the PC1/PC2 score plot (Figure 3a) it is clear that along with the PC1 component, there is a separation of the first group of objects which consists of **S1**, **S2**, **S3**, **S4**, **S9**, **S10** and the second group of objects which consists of **S5**, **S6**, **S7**, **S8**, **S11**, **S12** (Figure 3a). The greatest negative impact on the separation of the first group of objects along the PC 1 axis had Cd, Si, and Ti (Figure 3b), which is in line with the fact that the concentration of these elements in the samples **S1**, **S2**, **S3**, **S4**, **S9**, **S10** is lower compared to the concentration of these elements in the samples belonging to another group (Figure 3b). Mg and Cu have the most positive effect along the PC 1 axis on the separation of the samples. Other minerals (Ni, Al, Ba, Ca, Cr, Fe, Cu, Mn, Sr, Zn, S, K) have a higher concentration in the samples of the second group of objects compared to the samples from the first group of objects, so they are the reason for their separation.

Cluster analysis is a method for dividing a group of objects into classes so that similar objects are in the same class. Cluster analysis searches for objects which are close together in the variable space. The method of cluster analysis described here is hierarchical, meaning that once an object has been assigned to a group, the process cannot be reversed. HCA was performed in order to confirm the grouping of compounds already obtained by the PCA. It is sometimes used to make the process of grouping similar objects in PCA score graphs easier [67].

**Figure 4:**
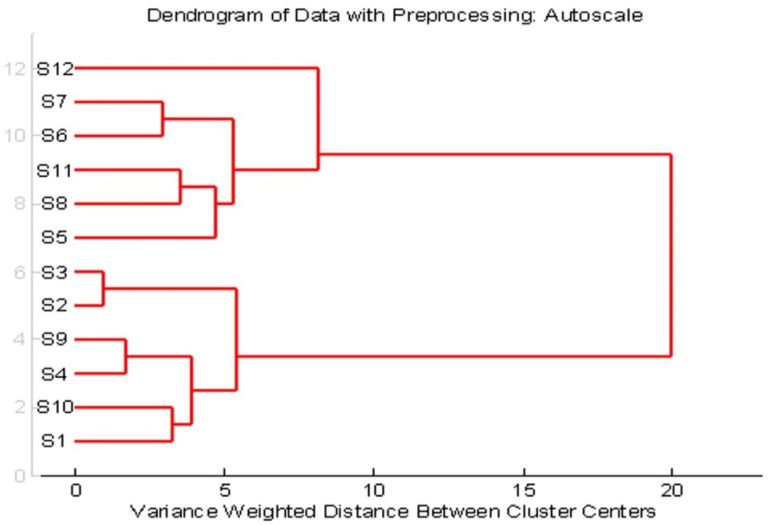
Dendrogram of chocolate samples with different cocoa content.

The obtained dendrogram based on spectrophotometry parameters shows two well-separated clusters (Figure 4). According to the PCA, separating the samples into two groups of objects was not the clearest, which is why the Hierarchical Cluster Analysis (HCA) analysis was performed. The results of the analysis confirmed the results of the PCA analysis. At a distance of 10, HCA resulted in the separation of the samples into two clusters, Figure 4. The first cluster includes the chocolate samples that have a lower percentage of cocoa or have added peppers, oranges, or are gluten-free, while the second cluster consists of the samples of collocations that have a higher percentage of cocoa and those with a high cocoa content and orange and raspberry content.

## 4. Conclusions

Chocolate samples with higher cocoa content were richer in total polyphenols and flavonoids. Antioxidant activities of chocolate samples measured through DPPH radical scavenging and FRAP assays showed a similar trend. The samples with the highest cocoa content had the highest activity applying DPPH and FRAP assays, whereas the highest total antioxidant capacity was observed for the samples with 99, 88, and 49% of cocoa. The highest content of Ca, K, Mg, S, Fe, Cu, and Zn was measured in the chocolate with 99% of cocoa, which can be attributed to the natural abundance of these elements in cocoa. The dietary intake of Mg, Fe, Mn, and Cu from 100 g of most dark chocolate samples compared with nutritional reference values was notable. On the other hand, the concentration of Cd was within the safe limits, whereas other toxic and potentially toxic elements were below the method quantification limits (MQL) in all the studied samples.

The present study provided valuable information on the quality of commercial dark chocolate in terms of polyphenol and flavonoid content as well as their antioxidant activity. Additionally, it offered an insight into the nutritionally significant elements in these prized products. By consuming dark chocolate with a high cocoa content, the body can be supplied with significant amounts of bioactive compounds, as well as essential macro- and microelements, which are proven to have positive effects on human health. Accordingly, and based on the results of this study, the consumption of dark chocolate, especially that with a high cocoa content, is recommended.

## Figures and Tables

**Table 1:** Descriptive statistics obtained from total polyphenol and total flavonoid content and antioxidant assays of dark chocolate samples.

	Sample	S1	S2	S3	S4	S5	S6	S7	S8	S9	S10	S11	S12
	Mean ± SD ^1^	10.55 ± 0.05 ^h^	15.74 ± 0.15 ^g^	15.07 ± 0.06 ^g^	15.30 ± 0.19 ^g^	14.97 ± 0.16 ^g^	17.61 ± 0.08 ^f^	19.63 ± 0.57 ^e^	25.38 ± 0.57 ^d^	29.58 ± 0.61 ^c^	28.76 ± 0.50 ^c^	35.96 ± 0.81 ^b^	39.82 ± 0.61 ^a^
	Median	10.55	15.71	15.10	15.33	15.02	17.61	19.80	25.45	29.73	29.00	36.09	40.10
**TPC**	Min ^2^	10.50	15.60	15.00	15.10	14.80	17.54	19.00	24.76	28.90	28.18	35.10	39.12
	Max ^3^	10.60	15.90	15.10	15.47	15.10	17.69	20.10	25.93	30.10	29.10	36.70	40.25
	Mean ± SD	10.04 ± 0.14 ^h^	15.46 ± 0.11 ^e,f^	14.92 ± 0.24 ^f,g^	15.13 ± 0.16 ^e,f,g^	13.72 ± 0.25 ^g^	16.11 ± 0.19 ^e,f^	16.45 ± 1.25 ^e^	26.68 ± 0.61 ^d^	31.59 ± 0.52 ^c^	30.99 ± 0.62 ^c^	34.85 ± 0.28 ^b^	37.85 ± 0.18 ^a^
	Median	10.10	15.50	15.00	15.10	13.67	16.16	15.97	26.86	31.55	31.10	34.90	37.80
**TFC**	Min	9.88	15.34	14.65	14.99	13.50	15.90	15.50	26.00	31.10	30.33	34.55	37.70
	Max	10.15	15.54	15.10	15.30	13.99	16.28	17.87	27.17	32.13	31.55	35.10	38.05
	Mean ± SD	29.61 ± 0.70 ^f^	32.74 ± 0.85 ^e^	35.07 ± 0.34 ^d,e^	38.06 ± 0.23 ^c^	38.74 ± 0.90 ^c^	38.17 ± 0.16 ^c^	37.19 ± 0.69 ^c,d^	42.69 ± 0.96 ^b^	42.11 ± 0.12 ^b^	44.58 ± 0.48 ^b^	47.47 ± 2.12 ^a^	48.34 ± 0.99 ^a^
	Median	30.00	32.55	35.00	37.98	38.55	38.21	37.55	42.88	42.10	44.50	46.55	48.17
**DPPH**	Min	28.80	32.01	34.76	37.88	37.95	38.00	36.39	41.65	42.00	44.15	45.97	47.44
	Max	30.04	33.67	35.44	38.31	39.72	38.31	37.63	43.55	42.24	45.10	49.90	49.40
	Mean ± SD	20.50 ± 1.17 ^j^	30.56 ± 1.23 ^i^	32.52 ± 0.48 ^h,i^	34.06 ± 0.46 ^g,h^	32.42 ± 0.56 ^h,i^	36.37 ± 0.89 ^g^	48.53 ± 0.57 ^f^	53.68 ± 0.86 ^e^	67.22 ± 1.11 ^d^	69.69 ± 0.38 ^c^	78.89 ± 0.97 ^b^	89.00 ± 0.57 ^a^
	Median	20.14	30.00	32.55	34.16	32.21	36.71	48.46	53.98	67.03	69.77	78.44	89.05
**FRAP**	Min	19.55	29.70	32.03	33.56	32.00	35.36	48.00	52.71	66.22	69.28	78.23	88.41
	RSD	5.69	4.04	1.46	1.36	1.71	2.45	1.17	1.60	1.65	0.55	1.23	0.64
	Mean ± SD	61.15 ± 1.26 ^c^	81.76 ± 3.03 ^a^	69.86 ± 1.04 ^b^	46.33 ± 0.39 ^e,f^	43.54 ± 1.34 ^f^	42.24 ± 0.40 ^f^	49.38 ± 1.21 ^d^	54.64 ± 0.98 ^c^	72.53 ± 1.01 ^b^	70.39 ± 1.55 ^b^	79.14 ± 0.99 ^a^	83.86 ± 1.30 ^a^
	Median	60.44	80.44	69.25	46.23	43.66	42.13	50.01	55.22	73.01	71.41	79.80	84.13
**TAC**	Min	60.10	78.88	69.00	45.90	41.85	41.81	47.68	53.27	71.12	68.21	77.74	82.15
	Max	62.92	85.95	71.33	46.85	45.12	42.77	50.44	55.44	73.46	71.56	79.87	85.31

^1^ Standard Deviation ^2^ Min -Minimum values; ^3^ Max—Maximum values; Data for all measurements made in triplicate are expressed as the mean ± standard deviation (SD).; ^a–h^ Different letters in the same column denote a significant difference according to Tukey’s test, *p* < 0.05. Parameters: TPC—Total polyphenol content; TFC—Total flavonoid content; DPPH-1,1-diphenyl-2-picryl-hydrazyl free radical scavenging assay (% of inhibition); FRAP—Ferric Reducing Antioxidant Power (mg/g GAE); TAC—Total Antioxidant Capacity (mg/g AAC). Samples and cocoa content: **S1**—40% with pepper, **S2**—49%, **S3**—56%, **S4**—73%, **S5**—75%, **S6**—75% with orange, **S7**—75% with raspberry, **S8**—77%, **S9**—77% with orange, **S10**—85% (gluten-free, no added sugar), **S11**—88%, **S12**—99%.

**Table 2:** Correlation coefficients obtained for the relationships between total polyphenols, total flavonoids, and antioxidant assays.

	**TPC**	**TFC**	**DPPH**	**FRAP**	**TAC**
**TPC**	1.00				
**TFC**	0.99	1.00			
**DPPH**	0.93	0.92	1.00		
**FRAP**	0.99	0.98	0.93	1.00	
**TAC**	0.58	0.60	0.32	0.55	1.00

TPC—Total Polyphenol Content; TFC—Total Flavonoid Content; DPPH -1,1-diphenyl-2-picryl-hydrazyl free radical scavenging assay (% of inhibition); FRAP—Ferric Reducing Antioxidant Power; TAC—Total Antioxidant Capacity.

**Table 3:** The number of principal components and the percentage of variance they explain.

Principal Component Number	Eigenvalue of Cov(X)	% Variance Captured	% Variance Captured Total
1	11.9	70.01	70.01
2	1.62	9.53	79.54
3	1.35	7.92	87.46

## Data Availability

Data is contained within the article or Supplementary Materials.

## Supplementary Materials

The following supporting information can be downloaded at: https://www.mdpi.com/article/10.3390/foods11101445/s1, Figure S1: Percentage of K, Ca and Mg dietary intake from 100 g of dark chocolate compared with nutritional reference values (NRV), Figure S2: Percentage of Fe, Mn, Cu and Zn dietary intake from 100 g of dark chocolate compared with nutritional reference values (NRV)*, Table S1: Producers and nutrition information (per 100 g) of analyzed chocolate samples, Table S2: Method parameters, Table S3: Method detection limits (MDL) and method quantification limits (MQL) for elements, Table S4: Parameters of descriptive statistics for elemental profile of chocolate with different cocoa content, Table S5: Present study Vs. literature data for elemental composition of dark chocolate, Table S6: Parameters of post-hoc Fisher test—LSD [6,25,26,27,28,29,30,31,32,45,65].
