# Supplementary material for: Antioxidant Activity and Multi-Elemental Analysis of Dark Chocolate
Source: Foods. 2022 May 17;11(10):1445. doi: 10.3390/foods11101445 (PMC9141620; doi:10.3390/foods11101445)
Supplement: Supplementary file 1 [file foods-11-01445-s001.zip › foods-1703453-supplementary.pdf]

# Supplementary Materials

**Table S1.** Producers and nutrition information (per 100 g) of analyzed chocolate samples

| Sample* | Brand | Energy value (cal) | Fat (g) | Carbohydrate (g) | Protein (g) |
|---|---|---|---|---|---|
| **S1** | Lindt & Sprüngl, Switzerland | 475 | 32 | 60 | 5 |
| **S2** | Maxi Premia, Serbia | 528 | 31.8 | 51.1 | 4.9 |
| **S3** | Roshen, Ukraine | 547 | 36.2 | 45.1 | 5.8 |
| **S4** | Hachez, Germany | 578 | 47 | 23 | 8.8 |
| **S5** | Soko-Štark d.o.o., Serbia | 552 | 40 | 33 | 10 |
| **S6** | Soko-Štark d.o.o., Serbia | 519 | 34 | 39 | 8.3 |
| **S7** | Soko-Štark d.o.o., Serbia | 540 | 38 | 35 | 9.5 |
| **S8** | Hachez, Germany | 565 | 45 | 24 | 8 |
| **S9** | Hachez, Germany | 570 | 46 | 25 | 9 |
| **S10** | Valor Chocolates S.A., Spain | 568 | 49 | 19 | 11 |
| **S11** | Hachez, Germany | 648 | 58 | 15 | 10 |
| **S12** | Lindt & Sprüngl, Switzerland | 573 | 49 | 10 | 14 |

* **S1** (40% cocoa with the addition of dried chili pepper), **S2** (49% cocoa), **S3** (56% cocoa), **S4** (73% cocoa), **S5** (75% cocoa), **S6** (75% cocoa with addition of dried orange), **S7** (75% cocoa with addition of dried raspberry), **S8** (77% cocoa), **S9** (77% cocoa with addition of orange), **S10** (85% cocoa, gluten-free, no sugar added), **S11** (88% cocoa), **S12** (99% cocoa).

**Table S2.** Method parameters.

| View Direction | Radial | Axial |
|---|---|---|
| UV Exposure time | 15 | 15 |
| UV RF Power | 1150 | 1150 |
| UV Neb Gas Flow | 0.5 | 0.5 |
| VIS Exposure Time | 5 | 5 |
| VIS RF Power | 1150 | 1150 |
| VIS Neb Gas Flow | 0.5 | 0.5 |
| Cool Gas Flow Rate | 12 | 12 |
| Aux Gas Flow Rate | 0.5 | 0.5 |

**Table S3.** Method detection limits (MDL) and method quantification limits (MQL) for elements

| | MQL (mg/kg) | MDL (mg/kg) |
|---|---|---|
| Sb | 0.224 | 0.067 |
| As | 0.580 | 0.174 |
| Cd | 0.032 | 0.010 |
| Co | 0.420 | 0.126 |
| Ni | 0.126 | 0.038 |
| Se | 0.448 | 0.134 |
| Tl | 0.364 | 0.109 |
| V | 0.182 | 0.055 |
| Al | 0.559 | 0.168 |
| Ba | 0.014 | 0.004 |
| B | 0.308 | 0.092 |
| Ca | 0.699 | 0.210 |
| Cr | 0.112 | 0.034 |
| Fe | 0.210 | 0.063 |
| Pb | 1.483 | 0.445 |
| Cu | 0.448 | 0.134 |

| | | |
|---|---|---|
| Mg | 0.056 | 0.017 |
| Mn | 0.014 | 0.004 |
| Si | 0.280 | 0.084 |
| Sr | 0.014 | 0.004 |
| Ti | 0.070 | 0.021 |
| Zn | 0.406 | 0.122 |
| S | 4.895 | 1.470 |
| Mo | 0.117 | 0.035 |
| Na | 3.636 | 1.092 |
| K | 98.643 | 29.623 |

**Table S4.** Parameters of descriptive statistics for elemental profile of chocolate with different cocoa content

| Sample | | Al | Ba | Ca | Cd | Co | Cr | Cu | Fe | K | Mg | Mn | Ni | S | Si | Sr | Ti | Zn |
|---|---|---|---|---|---|---|---|---|---|---|---|---|---|---|---|---|---|---|
| S1 | Mean | 20.54 | 2.11 | 491.86 | 0.03 | 0.36 | 0.91 | 7.73 | 224.85 | 1153.80 | 904.54 | 11.28 | 2.43 | 1249.28 | 3.17 | 3.70 | 0.96 | 13.39 |
| | St. Dev. | 1.33 | 0.05 | 2.67 | 0.00 | 0.00 | 0.01 | 0.02 | 1.93 | 4.53 | 2.11 | 0.09 | 0.00 | 4.00 | 0.05 | 0.02 | 0.06 | 0.35 |
| | Min | 19.31 | 2.06 | 489.76 | 0.03 | 0.36 | 0.90 | 7.72 | 223.18 | 1149.52 | 902.25 | 11.19 | 2.43 | 1245.36 | 3.11 | 3.69 | 0.90 | 13.12 |
| | Max | 21.95 | 2.14 | 494.87 | 0.03 | 0.36 | 0.92 | 7.75 | 226.97 | 1158.54 | 906.41 | 11.37 | 2.43 | 1253.36 | 3.22 | 3.72 | 1.01 | 13.79 |
| | Median | 20.35 | 2.14 | 490.95 | 0.03 | 0.36 | 0.90 | 7.73 | 224.40 | 1153.33 | 904.95 | 11.28 | 2.43 | 1249.11 | 3.18 | 3.70 | 0.97 | 13.26 |
| | RSD | 6.45 | 2.19 | 0.54 | 0.00 | 0.05 | 1.56 | 0.20 | 0.86 | 0.39 | 0.23 | 0.80 | 0.00 | 0.32 | 1.64 | 0.52 | 6.14 | 2.63 |
| S2 | Mean | 12.00 | 1.69 | 337.66 | 0.03 | 0.19 | 0.86 | 6.85 | 110.94 | 694.75 | 663.48 | 7.58 | 1.92 | 1058.68 | 1.62 | 3.74 | 0.23 | 12.15 |
| | St. Dev. | 0.65 | 0.06 | 1.26 | 0.00 | 0.01 | 0.01 | 0.02 | 1.00 | 4.65 | 2.07 | 0.10 | 0.01 | 4.50 | 0.03 | 0.03 | 0.04 | 0.25 |
| | Min | 11.35 | 1.62 | 336.46 | 0.03 | 0.18 | 0.86 | 6.83 | 109.95 | 689.87 | 661.21 | 7.51 | 1.92 | 1054.25 | 1.59 | 3.71 | 0.19 | 11.95 |
| | Max | 12.65 | 1.73 | 338.97 | 0.03 | 0.20 | 0.88 | 6.87 | 111.94 | 699.12 | 665.27 | 7.69 | 1.93 | 1063.25 | 1.65 | 3.77 | 0.27 | 12.43 |
| | Median | 12.02 | 1.73 | 337.56 | 0.03 | 0.18 | 0.86 | 6.87 | 110.92 | 695.25 | 663.96 | 7.52 | 1.92 | 1058.55 | 1.62 | 3.73 | 0.22 | 12.08 |
| | RSD | 5.41 | 3.80 | 0.37 | 0.00 | 6.13 | 1.14 | 0.36 | 0.90 | 0.67 | 0.31 | 1.34 | 0.32 | 0.43 | 1.89 | 0.84 | 19.20 | 2.02 |
| S3 | Mean | 11.38 | 1.99 | 356.14 | 0.03 | 0.20 | 0.68 | 6.70 | 166.19 | 769.01 | 717.95 | 8.95 | 1.90 | 1054.35 | 0.68 | 3.82 | 0.18 | 13.25 |
| | St. Dev. | 0.79 | 0.04 | 0.70 | 0.00 | 0.00 | 0.01 | 0.02 | 1.07 | 8.16 | 1.99 | 0.03 | 0.00 | 3.46 | 0.07 | 0.07 | 0.02 | 0.26 |
| | Min | 10.75 | 1.94 | 355.50 | 0.03 | 0.20 | 0.67 | 6.68 | 165.16 | 761.25 | 715.99 | 8.91 | 1.90 | 1051.66 | 0.60 | 3.79 | 0.16 | 13.03 |
| | Max | 12.25 | 2.01 | 356.89 | 0.03 | 0.20 | 0.69 | 6.73 | 167.30 | 777.52 | 719.97 | 8.98 | 1.90 | 1058.26 | 0.75 | 3.87 | 0.20 | 13.53 |
| | Median | 11.12 | 2.01 | 356.05 | 0.03 | 0.20 | 0.68 | 6.71 | 166.13 | 768.25 | 717.90 | 8.95 | 1.90 | 1053.14 | 0.68 | 3.81 | 0.18 | 13.20 |
| | RSD | 6.90 | 2.03 | 0.20 | 0.00 | 0.17 | 1.67 | 0.32 | 0.64 | 1.06 | 0.28 | 0.36 | 0.13 | 0.33 | 10.61 | 1.88 | 11.81 | 1.95 |
| S4 | Mean | 15.51 | 2.63 | 437.46 | 0.13 | 0.30 | 0.29 | 7.41 | 155.55 | 1362.00 | 906.90 | 8.48 | 1.97 | 1407.68 | 0.28 | 5.17 | 1.24 | 18.76 |
| | St. Dev. | 0.53 | 0.02 | 0.96 | 0.00 | 0.00 | 0.01 | 0.03 | 1.06 | 7.43 | 3.04 | 0.06 | 0.03 | 3.49 | 0.00 | 0.06 | 0.04 | 0.16 |
| | Min | 14.95 | 2.61 | 436.45 | 0.13 | 0.30 | 0.28 | 7.38 | 154.60 | 1355.11 | 903.84 | 8.41 | 1.95 | 1404.25 | 0.28 | 5.12 | 1.20 | 18.62 |
| | Max | 16.00 | 2.65 | 438.36 | 0.13 | 0.30 | 0.30 | 7.44 | 156.70 | 1369.88 | 909.92 | 8.52 | 2.00 | 1411.23 | 0.28 | 5.24 | 1.27 | 18.93 |
| | Median | 15.57 | 2.62 | 437.58 | 0.13 | 0.30 | 0.30 | 7.42 | 155.36 | 1361.02 | 906.93 | 8.49 | 1.95 | 1407.56 | 0.28 | 5.17 | 1.24 | 18.73 |
| | RSD | 3.39 | 0.66 | 0.22 | 1.57 | 0.08 | 3.44 | 0.34 | 0.68 | 0.55 | 0.34 | 0.68 | 1.32 | 0.25 | 0.00 | 1.15 | 2.85 | 0.84 |

**Table S4. - Continued**

| Sample | | Al | Ba | Ca | Cd | Co | Cr | Cu | Fe | K | Mg | Mn | Ni | S | Si | Sr | Ti | Zn |
|---|---|---|---|---|---|---|---|---|---|---|---|---|---|---|---|---|---|---|
| **S5** | Mean | 36.19 | 3.04 | 673.06 | 0.24 | 0.43 | 1.90 | 11.92 | 371.90 | 1703.01 | 1301.12 | 16.15 | 3.76 | 2121.48 | 1.23 | 6.55 | 1.52 | 25.03 |
| | St. Dev. | 0.71 | 0.06 | 1.30 | 0.00 | 0.00 | 0.02 | 1.30 | 1.07 | 5.76 | 2.67 | 0.25 | 0.00 | 3.03 | 0.07 | 0.04 | 0.07 | 0.32 |
| | Min | 35.46 | 2.98 | 671.59 | 0.24 | 0.43 | 1.88 | 11.88 | 370.82 | 1698.33 | 1298.25 | 15.88 | 3.76 | 2118.15 | 1.17 | 6.51 | 1.45 | 24.71 |
| | Max | 36.88 | 3.11 | 674.07 | 0.24 | 0.43 | 1.92 | 11.94 | 372.95 | 1709.44 | 1303.53 | 16.38 | 3.77 | 2124.06 | 1.30 | 6.60 | 1.59 | 25.36 |
| | Median | 36.23 | 3.04 | 673.53 | 0.24 | 0.43 | 1.91 | 11.92 | 371.94 | 1701.25 | 1301.56 | 16.19 | 3.77 | 2122.24 | 1.23 | 6.55 | 1.52 | 25.02 |
| | RSD | 1.97 | 2.10 | 0.19 | 0.25 | 0.04 | 0.93 | 10.94 | 0.29 | 0.34 | 0.20 | 1.57 | 0.12 | 0.14 | 5.56 | 0.69 | 4.50 | 1.28 |
| **S6** | Mean | 25.10 | 3.72 | 908.06 | 0.04 | 0.35 | 1.19 | 10.82 | 157.05 | 1296.68 | 1105.20 | 16.16 | 3.37 | 1777.89 | 2.12 | 8.67 | 0.08 | 20.38 |
| | St. Dev. | 0.34 | 0.01 | 1.42 | 0.00 | 0.00 | 0.01 | 0.03 | 1.27 | 5.03 | 2.48 | 0.36 | 0.01 | 4.04 | 0.09 | 0.05 | 0.00 | 0.44 |
| | Min | 24.77 | 3.70 | 906.46 | 0.04 | 0.35 | 1.19 | 10.78 | 155.99 | 1291.55 | 1102.45 | 15.79 | 3.36 | 1773.55 | 2.02 | 8.62 | 0.07 | 19.95 |
| | Max | 25.46 | 3.72 | 909.17 | 0.04 | 0.35 | 1.20 | 10.85 | 158.46 | 1301.61 | 1107.26 | 16.51 | 3.38 | 1781.55 | 2.19 | 8.72 | 0.08 | 20.83 |
| | Median | 25.05 | 3.72 | 908.55 | 0.04 | 3.72 | 1.20 | 10.82 | 156.70 | 1296.88 | 1105.90 | 16.17 | 3.38 | 1778.58 | 2.17 | 8.68 | 0.08 | 20.35 |
| | RSD | 1.37 | 0.34 | 0.16 | 5.79 | 0.27 | 0.62 | 0.29 | 0.81 | 0.39 | 0.22 | 2.23 | 0.37 | 0.23 | 4.44 | 0.60 | 5.88 | 2.16 |
| **S7** | Mean | 25.57 | 4.05 | 801.92 | 0.04 | 0.43 | 1.01 | 12.19 | 212.01 | 1635.88 | 1229.33 | 21.52 | 3.74 | 2150.00 | 0.58 | 8.39 | 1.16 | 22.17 |
| | St. Dev. | 0.37 | 0.06 | 1.65 | 0.00 | 0.00 | 0.02 | 0.05 | 1.01 | 5.79 | 1.77 | 0.07 | 0.02 | 6.60 | 0.08 | 0.09 | 0.05 | 0.16 |
| | Min | 25.31 | 3.99 | 800.96 | 0.04 | 0.43 | 0.99 | 12.13 | 210.99 | 1630.25 | 1227.76 | 21.45 | 3.73 | 2145.55 | 0.49 | 8.33 | 1.11 | 22.01 |
| | Max | 26.00 | 4.10 | 803.83 | 0.04 | 0.43 | 1.03 | 12.24 | 213.00 | 1641.82 | 1231.25 | 21.59 | 3.77 | 2157.58 | 0.66 | 8.49 | 1.21 | 22.32 |
| | Median | 25.39 | 4.07 | 800.97 | 0.04 | 0.43 | 1.00 | 12.20 | 212.05 | 1635.58 | 1229.00 | 21.53 | 3.73 | 2146.87 | 0.59 | 8.35 | 1.15 | 22.17 |
| | RSD | 1.46 | 1.53 | 0.21 | 2.55 | 0.14 | 2.02 | 0.44 | 0.47 | 0.35 | 0.14 | 0.33 | 0.62 | 0.31 | 14.45 | 1.09 | 4.35 | 0.70 |
| **S8** | Mean | 17.05 | 3.78 | 601.65 | 0.25 | 0.34 | 0.86 | 10.44 | 183.85 | 1154.97 | 1085.83 | 10.52 | 2.13 | 1833.38 | 2.51 | 7.68 | 0.74 | 24.61 |
| | St. Dev. | 0.17 | 0.03 | 1.06 | 0.00 | 0.00 | 0.02 | 0.03 | 0.91 | 4.87 | 2.61 | 0.07 | 0.02 | 3.87 | 0.08 | 0.10 | 0.05 | 0.22 |
| | Min | 16.89 | 3.74 | 600.65 | 0.25 | 0.34 | 0.84 | 10.40 | 182.89 | 1151.22 | 1082.88 | 10.47 | 2.10 | 1829.48 | 2.43 | 7.61 | 0.69 | 24.39 |
| | Max | 17.24 | 3.80 | 602.77 | 0.25 | 0.34 | 0.88 | 10.47 | 184.71 | 1160.47 | 1087.81 | 10.60 | 2.14 | 1837.21 | 2.60 | 7.80 | 0.79 | 24.83 |
| | Median | 17.03 | 3.80 | 601.55 | 0.25 | 0.34 | 0.85 | 10.45 | 183.95 | 1153.21 | 1086.80 | 10.50 | 2.14 | 1833.44 | 2.51 | 7.64 | 0.74 | 24.60 |
| | RSD | 1.02 | 0.84 | 0.18 | 0.44 | 0.14 | 1.98 | 0.31 | 0.50 | 0.42 | 0.24 | 0.64 | 0.92 | 0.21 | 3.31 | 1.29 | 6.69 | 0.90 |

**Table S4. - Continued**

| Sample | | Al | Ba | Ca | Cd | Co | Cr | Cu | Fe | K | Mg | Mn | Ni | S | Si | Sr | Ti | Zn |
|---|---|---|---|---|---|---|---|---|---|---|---|---|---|---|---|---|---|---|
| **S9** | Mean | 15.03 | 2.52 | 500.00 | 0.20 | 0.25 | 0.07 | 7.19 | 56.90 | 952.28 | 850.18 | 6.90 | 1.62 | 1300.69 | 0.28 | 5.49 | 0.98 | 15.85 |
| | St. Dev. | 0.11 | 0.01 | 1.09 | 0.00 | 0.00 | 0.01 | 0.03 | 0.01 | 6.25 | 2.98 | 0.06 | 0.01 | 2.86 | 0.00 | 0.07 | 0.07 | 0.15 |
| | Min | 14.90 | 2.51 | 498.90 | 0.20 | 0.25 | 0.07 | 7.16 | 56.89 | 945.52 | 847.20 | 6.85 | 1.61 | 1298.58 | 0.28 | 5.41 | 0.91 | 15.69 |
| | Max | 15.10 | 2.54 | 501.07 | 0.20 | 0.25 | 0.08 | 7.22 | 56.91 | 957.85 | 853.16 | 6.96 | 1.63 | 1303.94 | 0.28 | 5.56 | 1.05 | 15.99 |
| | Median | 15.08 | 2.52 | 500.05 | 0.20 | 0.25 | 0.08 | 7.19 | 56.90 | 953.47 | 850.19 | 6.89 | 1.61 | 1299.54 | 0.28 | 5.49 | 0.98 | 15.87 |
| | RSD | 0.74 | 0.48 | 0.22 | 0.28 | 0.31 | 10.91 | 0.47 | 0.01 | 0.66 | 0.35 | 0.85 | 0.83 | 0.22 | 0.00 | 1.35 | 7.31 | 0.94 |
| **S10** | Mean | 14.93 | 2.18 | 502.65 | 0.05 | 0.30 | 0.46 | 9.82 | 181.14 | 1222.70 | 1144.14 | 11.68 | 3.07 | 1805.69 | 0.28 | 5.93 | 0.32 | 20.45 |
| | St. Dev. | 0.16 | 0.03 | 1.41 | 0.00 | 0.00 | 0.02 | 0.03 | 0.96 | 4.73 | 3.73 | 0.08 | 0.02 | 5.04 | 0.00 | 0.06 | 0.04 | 0.25 |
| | Min | 14.76 | 2.15 | 501.15 | 0.05 | 0.30 | 0.45 | 9.80 | 180.25 | 1218.23 | 1140.22 | 11.59 | 3.06 | 1801.55 | 0.28 | 5.88 | 0.28 | 20.20 |
| | Max | 15.06 | 2.20 | 503.95 | 0.05 | 0.30 | 0.48 | 9.85 | 182.15 | 1227.65 | 1147.66 | 11.76 | 3.09 | 1811.31 | 0.28 | 5.99 | 0.37 | 20.70 |
| | Median | 14.96 | 2.20 | 502.85 | 0.05 | 0.30 | 0.45 | 9.82 | 181.02 | 1222.22 | 1144.53 | 11.68 | 3.06 | 1804.22 | 0.28 | 5.92 | 0.33 | 20.45 |
| | RSD | 1.04 | 1.16 | 0.28 | 0.00 | 0.42 | 3.53 | 0.29 | 0.53 | 0.39 | 0.33 | 0.70 | 0.64 | 0.28 | 0.00 | 1.00 | 12.72 | 1.22 |
| **S11** | Mean | 24.60 | 3.94 | 584.13 | 0.30 | 0.56 | 0.43 | 11.27 | 188.64 | 1855.09 | 1200.81 | 12.34 | 3.78 | 1965.26 | 4.37 | 7.63 | 1.41 | 22.07 |
| | St. Dev. | 0.37 | 0.01 | 1.01 | 0.01 | 0.00 | 0.01 | 0.27 | 0.76 | 5.81 | 3.13 | 0.02 | 0.01 | 7.42 | 0.08 | 0.07 | 0.05 | 0.25 |
| | Min | 24.26 | 3.92 | 583.15 | 0.29 | 0.56 | 0.42 | 11.03 | 187.92 | 1849.58 | 1198.16 | 12.32 | 3.77 | 1958.86 | 4.29 | 7.57 | 1.37 | 21.87 |
| | Max | 24.99 | 3.95 | 585.16 | 0.30 | 0.56 | 0.43 | 11.56 | 189.43 | 1861.15 | 1204.26 | 12.36 | 3.78 | 1973.39 | 4.44 | 7.71 | 1.46 | 22.35 |
| | Median | 24.55 | 3.93 | 584.07 | 0.30 | 0.56 | 0.43 | 11.21 | 188.56 | 1854.53 | 1200.00 | 12.35 | 3.77 | 1963.54 | 4.38 | 7.63 | 1.42 | 22.00 |
| | RSD | 1.50 | 0.35 | 0.17 | 1.92 | 0.13 | 1.19 | 2.40 | 0.40 | 0.31 | 0.26 | 0.19 | 0.15 | 0.38 | 1.80 | 0.90 | 3.29 | 1.14 |
| **S12** | Mean | 23.64 | 4.36 | 751.13 | 0.12 | 0.53 | 2.63 | 17.14 | 478.95 | 2962.35 | 1564.86 | 20.18 | 4.93 | 3010.16 | 5.71 | 8.76 | 1.64 | 34.57 |
| | St. Dev. | 0.39 | 0.05 | 1.05 | 0.00 | 0.00 | 0.02 | 0.19 | 0.76 | 4.78 | 4.59 | 0.05 | 0.00 | 5.21 | 0.09 | 0.06 | 0.07 | 0.34 |
| | Min | 23.22 | 4.30 | 750.06 | 0.12 | 0.52 | 2.61 | 16.95 | 477.96 | 2958.09 | 1560.54 | 20.14 | 4.93 | 3004.66 | 5.62 | 8.70 | 1.59 | 34.23 |
| | Max | 24.00 | 4.40 | 752.15 | 0.12 | 0.53 | 2.65 | 17.33 | 480.03 | 2967.52 | 1569.68 | 20.24 | 4.93 | 3015.02 | 5.80 | 8.82 | 1.72 | 34.91 |
| | Median | 23.70 | 4.38 | 751.16 | 0.12 | 0.53 | 2.63 | 17.14 | 478.86 | 2961.43 | 1564.36 | 20.17 | 4.93 | 3010.81 | 5.72 | 8.76 | 1.63 | 34.56 |
| | RSD | 1.67 | 1.22 | 0.14 | 0.52 | 0.64 | 0.71 | 1.11 | 0.16 | 0.16 | 0.29 | 0.27 | 0.07 | 0.17 | 1.57 | 0.72 | 4.02 | 1.00 |

**Table S5.** Present study Vs. literature data for elemental composition of dark chocolate

| **Reference** | **Present study** | **[6]** | **[25]** | **[27]** | **[28]** | **[29]** | **[26‡,65&]** | **[30]** | **[31]** | **[32]** |
|---|---|---|---|---|---|---|---|---|---|---|
| * | **ICP-OES** | **ICP-MS** | | **ICP OES** and **ICP-MS§** | | **ICP-OES** | | **ICP-OES** | **ICP-OES** | **MIP-OES†** |
| ** | **43-99%** | **53-85%** | **50-70%** | **55-85%** | **60-90%** | **70-75%** | **‡ - 44-90%**<br>**& 50-100%** | **70-85%** | **40-90%** | **55-85%** |
| **Al** | 11.38-36.19 | | 9.9-30.1 | | 5.6-13.8 | 7.2-112 | | | 5.4-69 | |
| **Ba** | 1.69-4.36 | | 4.25-8.99 | | 5.4-8.0 | 2.90-6.85 | | | 2.03-6.28 | |
| **Ca** | 337.69-908.12 | | 449-1421 | 578–1157 | 643.3-908.3 | 118-884 | | 533.3-1027.4 | 319.9- 1318.9 | 653-903 |
| **Cd** | 0.03-0.3 | | 0.043-0.510 | | | 0.022-0.178 | 0.004-3.15& | | | |
| **Co** | 0.19-0.56 | | 0.286-0.668 | | 0.33-0.58 | 0.047-0.457 | | | | |
| **Cr** | 0.07-2.63 | 0.48 - 2.91 | 0.41-2.27 | | | 0.23-1.69 | | 0.1-0.2 | | 0.003-2.8 |
| **Cu** | 6.70-17.14 | | 8.5-26.7 | 7.6–13.6§ | 14.3-20.2 | 9.5-16.2 | 5.05-16.5‡ | 7.5-8.5 | 21.5-39.5 | 7.6-19.5 |
| **Fe** | 56.9-478.95 | | 45.7-162 | | 97.3-112.4 | 37.8-179 | | 28.9-33.1 | 99.41 – 331.7 | 57-227 |
| **K** | 695.3-2962.1 | | | 4613–7006 | 4655.5-7201.1 | 4950-12700 | | 3651.1-4209.9 | 381.9-1082.7 | 4885-8574 |
| **Mg** | 663.48-1564.86 | | 1104-2383 | 1268–1910 | 1587.8-2522.1 | 855-1590 | | 1416.7-1993.9 | 1348.4- 3622 | 1083-2775 |
| **Mn** | 6.9 - 21.52 | | 10.2-21.5 | 9.7–14.7§ | 16.5-20.5 | 7.90-16.2 | | 7.7-11.1 | 14.5-15.5 | 9.6-25.2 |
| **Ni** | 1.62-4.93 | | 2.57-7.93 | | 3.6-6.1 | 2.29-5.90 | | | 4.5-9.2 | 5.0-9.0 |
| **S** | 1053.6-3009.7 | | 669-1443 | | | | | | 417.77-639.52 | |
| **Si** | 0.28- 5.7 | | 27-100 | | | 8.2-219 | | | | |
| **Sr** | 3.70- 8.76 | | 5.74-7.81 | | 0.04-0.08 | 4.20-7.90 | | | 5.5-6.2 | |
| **Ti** | 0.08-1.64 | | 0.002-0.006 | | | | | | | |
| **Zn** | 12.15-34.6 | | 18.5-41.5 | 38.4–64.8§ | 22.4-35.2 | 16.3-28.9 | | 17.7-20.5 | 16.86-39.26 | 20.4-33 |

Concentrations are expressed as mg/kg

*Methods; Inductively coupled plasma optical emission spectrometry (ICP-OES); Inductively coupled plasma mass spectrometry (ICP-MS); Microwave-induced plasma optical emission spectrometry (MIP-OES)

**Cocoa percentage

**Table S6.** Parameters of post-hoc Fisher test - LSD

| Cd | S1 | S2 | S3 | S4 | S5 | S6 | S7 | S8 | S9 | S10 | S11 | S12 |
|---|---|---|---|---|---|---|---|---|---|---|---|---|
| **S1** | 0.0000 | 0.0000 | 0.0000 | 0.0984 | 0.2052 | 0.0059 | 0.0041 | 0.2148 | 0.1643 | 0.0140 | 0.2681 | 0.0912 |
| **S2** | 0.0000 | 0.0000 | 0.0000 | 0.0984 | 0.2052 | 0.0059 | 0.0041 | 0.2148 | 0.1643 | 0.0140 | 0.2681 | 0.0912 |
| **S3** | 0.0000 | 0.0000 | 0.0000 | 0.0984 | 0.2052 | 0.0059 | 0.0041 | 0.2148 | 0.1643 | 0.0140 | 0.2681 | 0.0912 |
| **S4** | 0.0984 | 0.0984 | 0.0984 | 0.0000 | 0.1067 | 0.0926 | 0.0943 | 0.1163 | 0.0659 | 0.0844 | 0.1696 | 0.0073 |
| **S5** | 0.2052 | 0.2052 | 0.2052 | 0.1067 | 0.0000 | 0.1993 | 0.2011 | 0.0096 | 0.0408 | 0.1912 | 0.0629 | 0.1140 |
| **S6** | 0.0059 | 0.0059 | 0.0059 | 0.0926 | 0.1993 | 0.0000 | 0.0018 | 0.2089 | 0.1585 | 0.0081 | 0.2622 | 0.0853 |
| **S7** | 0.0041 | 0.0041 | 0.0041 | 0.0943 | 0.2011 | 0.0018 | 0.0000 | 0.2107 | 0.1602 | 0.0099 | 0.2640 | 0.0871 |
| **S8** | 0.2148 | 0.2148 | 0.2148 | 0.1163 | 0.0096 | 0.2089 | 0.2107 | 0.0000 | 0.0504 | 0.2008 | 0.0533 | 0.1236 |
| **S9** | 0.1643 | 0.1643 | 0.1643 | 0.0659 | 0.0408 | 0.1585 | 0.1602 | 0.0504 | 0.0000 | 0.1503 | 0.1037 | 0.0732 |
| **S10** | 0.0140 | 0.0140 | 0.0140 | 0.0844 | 0.1912 | 0.0081 | 0.0099 | 0.2008 | 0.1503 | 0.0000 | 0.2541 | 0.0772 |
| **S11** | 0.2681 | 0.2681 | 0.2681 | 0.1696 | 0.0629 | 0.2622 | 0.2640 | 0.0533 | 0.1037 | 0.2541 | 0.0000 | 0.1769 |
| **S12** | 0.0912 | 0.0912 | 0.0912 | 0.0073 | 0.1140 | 0.0853 | 0.0871 | 0.1236 | 0.0732 | 0.0772 | 0.1769 | 0.0000 |
| *LSD | 0.0033 | | | | | | | | | | | |

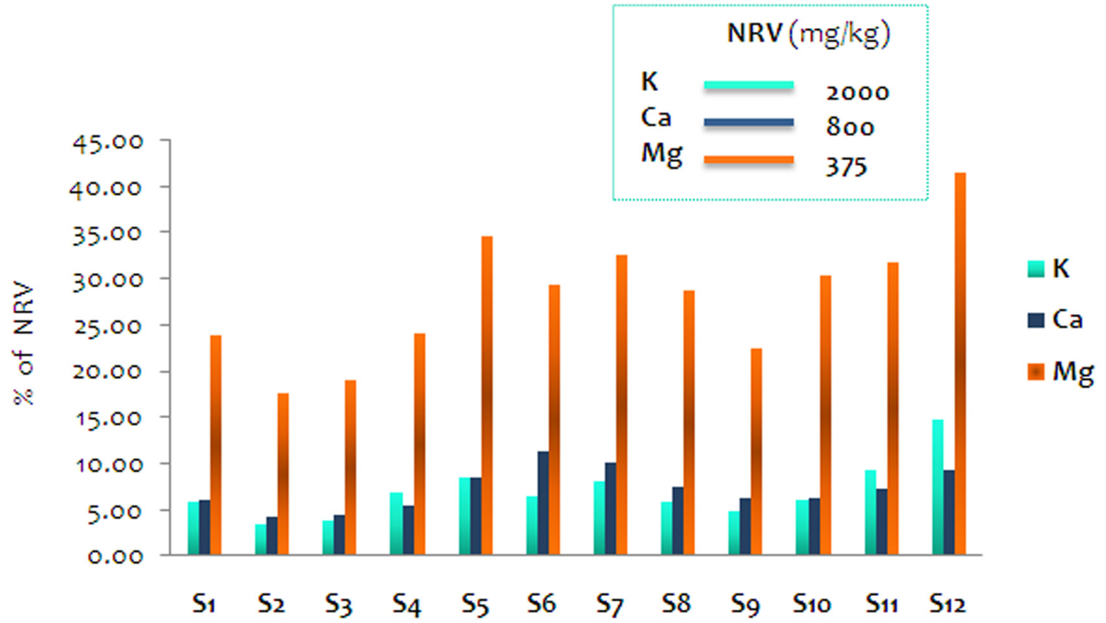


**Figure S1.** Percentage of K, Ca and Mg dietary intake from 100 g of dark chocolate compared with nutritional reference values (NRV)
(*NRV according to ref. [45])

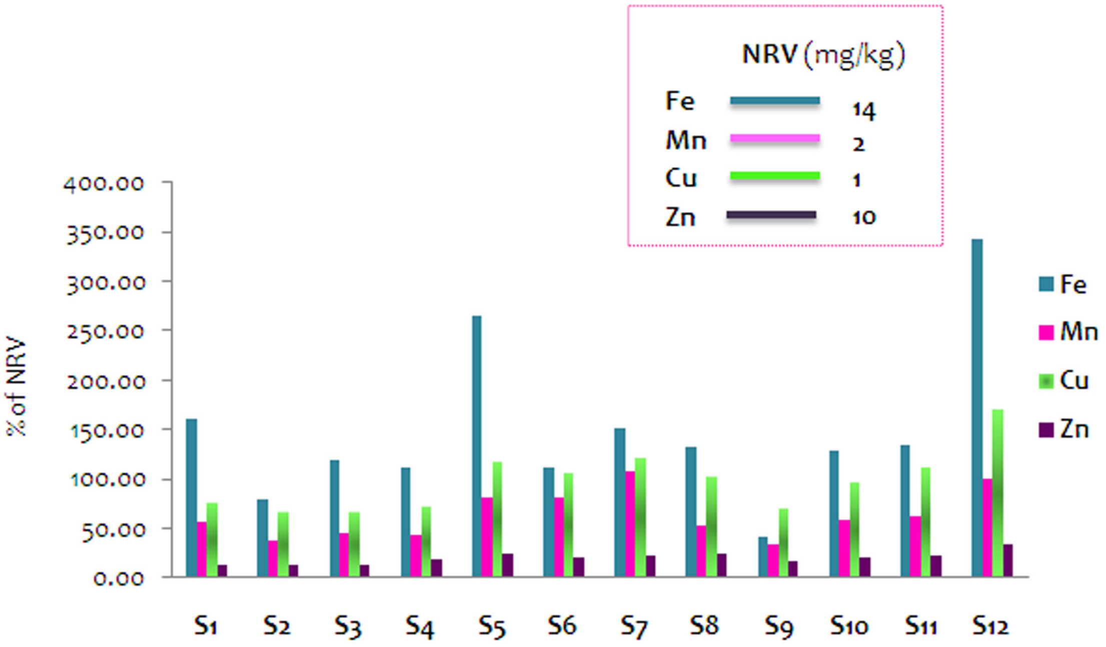


**Figure S2.** Percentage of Fe, Mn, Cu and Zn dietary intake from 100 g of dark chocolate compared with nutritional reference values (NRV)*
(*NRV according to ref. [45])

## References

[6] Yanus, R.L.; Sela, H.; Borojovich, E.J.C.; Zakon, Y.; Saphier, M.; Nikolski, A.; Gutflais, E.; Lorber, A.; Karpas, Z. Trace elements in cocoa solids and chocolate: An ICPMS study. *Talanta* **2014**, *119*, 1–4.

[25] Sager, M. Chocolate and cocoa products as a source of essential elements in nutrition. *J. Nutr. Food Sci.* **2012**, *2*, 123.

[26] Weber, T.; Solioz, M. Evaluation of chocolate as a source of dietary copper. *Eur. Food Res. Technol*. **2014**, *238*, 1063–1066.

[27] Villa, J.E.L.; Pereira, C.D.; Cadore, S. A novel, rapid and simple acid extraction for multielemental determination in chocolate bars. *Microchem. J.* **2015**, *121*, 199–204.

[28] Cinquanta, L.; Di Cesare, C.; Manoni, R.; Piano, A.; Roberti, P.; Salvatori, G. Mineral essential elements for nutrition in different chocolate products. *Int. J. Food Sci. Nutr.* **2016**, *67*, 773–778.

[29] Mrmošanin, M.J.; Pavlović, N.A.; Krstić, N.J.; Mitić, S.S.; Tošić, B.S.; Stojković, B.M.; Micić, J.R.; Đorđević, S.M. Multielemental quantification in dark chocolate by ICP OES. *J. Food Compos. Anal.* **2018**, *67*, 163–171.

[30] Grassia, M.; Salvatori, G.; Roberti, M.; Planeta, D.; Cinquanta, L. Polyphenols, methylxanthines, fatty acids and minerals in cocoa beans and cocoa products. *Food Measure.* **2019**, *13*, 1721–1728.

[31] Karaş, K.; Zioła-Frankowska, A; Bartoszewicz, M.; Krzyśko. G; Frankowski, M. Investigation of chocolate types on the content of selected metals and non-metals determined by ICP-OES analytical technique, *Food Addit. Contam. Part A*, **2021**, *38*, 293–303. DOI: 10.1080/19440049.2020.1853821

[32] Oliveira, L.B.; de Melo, J.C.; da Boa Morte, E.S.; de Jesus, R.M.; Teixeira, L.S.G.; Korn, G.A.M., Multi-element determination in chocolate bars by microwave-induced plasma optical emission spectrometry, *Food Chem*. **2021**, *351*, 129285. https://doi.org/10.1016/j.foodchem.2021.129285.

[45] Pravilnik o deklarisanju, označavanju i reklamiranju hrane ("Regulation on labelling and advertising of food") ("Official Gazette RS". No. 19/2017 and 16/2018) (in Serbian); http://demo.paragraf.rs/WebParagrafDemo/?did=424789. Accessed on 20 January 2022

[65] Abt, E.; Fong Sam, J.; Gray, P.; Posnick Robin, L. Cadmium and lead in cocoa powder and chocolate products in the US Market. *Food Addit. Contam.*: *B Surveill*. **2018**, *11*, 92–102. doi: 10.1080/19393210.2017.1420700
